# KILDA: identifying KIV-2 repeats from kmers

**DOI:** 10.1093/nargab/lqaf070

**Published:** 2025-05-30

**Authors:** Corentin Molitor, Timothy Labidi, Antoine Rimbert, Bertrand Cariou, Mathilde Di Filippo, Claire Bardel

**Affiliations:** Plateforme NGS-HCL, Cellule bioinformatique, Service de génétique, Hospices Civils de Lyon, 69000, Bron, France; Plateforme NGS-HCL, Cellule bioinformatique, Service de génétique, Hospices Civils de Lyon, 69000, Bron, France; Nantes Universit é, CNRS, CHU Nantes, Inserm, l’institut du thorax, 44000, Nantes, France; Nantes Universit é, CNRS, CHU Nantes, Inserm, l’institut du thorax, 44000, Nantes, France; Service de Biochimie et Biologie Moléculaire, Laboratoire de Biologie Médicale MultiSites, Hospices Civils de Lyon, 69000, Bron, France; CarMen Laboratory, INSERM, INRAE, U niversité Claude Bernard Lyon 1, 69500, Pierre-Bénite, France; Plateforme NGS-HCL, Cellule bioinformatique, Service de génétique, Hospices Civils de Lyon, 69000, Bron, France; Universite Claude Bernard Lyon 1, LBBE, UMR 5558, CNRS, 69100, Villeurbanne, France

## Abstract

High concentration of lipoprotein(a) [Lp(a)], a lipoprotein with proatherogenic properties, is an important risk factor for cardiovascular disease. This concentration is mostly genetically determined by a complex interplay between the number of kringle IV type 2 repeats and Lp(a)-affecting variants. Besides Lp(a) plasma concentration, there is an unmet need to identify individuals most at risk based on their *LPA* genotype. We developed KILDA (KIv2 Length Determined from a kmer Analysis), a Nextflow pipeline, to identify the number of kringle IV type 2 repeats and Lp(a)-affecting variants directly from kmers generated from FASTQ files. The pipeline was tested on the 1000 Genomes Project (*n* = 2459) and results were equivalent to DRAGEN-LPA (*R*^2^= 0.92). *In silico* datasets proved the robustness of KILDA’s predictions under different scenarios of sequencing coverage and quality. In brief, KILDA is a robust, open-source, and free-to-use pipeline that can identify the number of kringle IV type 2 repeats and Lp(a)-associated variants even when inputting low-coverage libraries.

## Introduction

Cardiovascular disease (CVD) is one of the leading causes of death worldwide and is on the rise [1]. Concentration of lipoprotein(a) [Lp(a)] in the blood is an independent and important risk factor for CVD [2, 3]. Lp(a) is composed of a low-density lipoprotein (LDL)-like particle in which apolipoprotein B (apoB) is bound to apolipoprotein(a) (apo[a]), the pathognomonic component of Lp(a). The Lp(a) concentration remains stable across the lifespan of an individual and is mostly genetically determined by the number of repeats of kringle IV type 2 (KIV-2) [4]. KIV-2 is a repeated region, ∼5.5 kb long, with a highly variable number of copies between individuals. Importantly, the number of KIV-2 repeats is inversely correlated with Lp(a) plasma levels [5]. This relationship is impacted by variants altering Lp(a) levels, which can mask the effect of apo[a] isoforms because of linkage between these variants and KIV-2 repeats, highlighting the importance of independently measuring genetic KIV-2 repeats. This relationship is further complexified by the existence of null alleles and variant–variant interactions [4]. Despite recommendations from the European and French societies [3, 6], Lp(a) measurement is not performed routinely in clinical practice, neither systematically nor in subjects at high cardiovascular risk. This is mainly due to the fact that medical laboratories are not equipped to measure Lp(a) and that such analyses are costly and not necessarily reimbursed.

Moreover, the diagnosis of familial hypercholesterolemia (FH) is characterized by genetically defined high levels of LDL-cholesterol. LDL-cholesterol plasma levels are, in most cases, calculated from formulas using total cholesterol [7]. The cholesterol content of Lp(a) can interfere with these equations [8]. Consequently, individuals with elevated Lp(a) levels may be erroneously diagnosed with FH and could lead to unnecessary FH genetic testing [9]. When these tests yield negative results, the subsequent cascade screening, a process to identify affected family members, is not initiated. This oversight, in light of coming Lp(a)-lowering drugs [10], may result in missed opportunities for early detection and intervention in truly affected individuals and their relatives.

An open question remains: How can we efficiently detect, using a genetic approach, hypercholesterolemic patients most likely to have elevated Lp(a) concentration? As Lp(a) concentrations are highly heritable, leveraging latent genetic information provides a promising approach to pinpoint these patients. For this purpose, the *LPA* gene and variants that can predict circulating concentrations of Lp(a) [11] must be sequenced, and a tool is required to accurately estimate the KIV-2 copy numbers. Recently, the DRAGEN-LPA caller [12] managed to get an accurate estimation of KIV-2 copy numbers. However, DRAGEN-LPA has been developed to process whole genome sequencing data, which is not ideal to use in molecular diagnosis, due to costs and closed-source code limitations.

Here, we present KILDA (KIv2 Length Determined from a kmer Analysis), an open-source and freely available pipeline, written in Nextflow and Python. KILDA is designed to estimate KIV-2 copy numbers and detect the presence of Lp(a)-affecting variants directly from FASTQ files. We benchmarked our tool against DRAGEN-LPA using the 1000 Genomes Project dataset. Additionally, we evaluated KILDA’s accuracy using simulated samples with predetermined KIV-2 repeat counts, testing its robustness under various constraints of sequencing coverage and quality.

## Materials and methods

KILDA estimates KIV-2 repeat numbers by analyzing the ratio of kmer occurrences between the KIV-2 region and one or more normalization regions. The underlying principle is that individuals with a higher number of KIV-2 repeats will exhibit higher occurrences of KIV-2 kmers. The normalization regions are single-copy genomic areas, serving as a calibration factor to account for variations in sequencing depth across individuals. The *LPA* gene (excluding KIV-2) was used for the analyses described in this paper, but the pipeline is flexible and can accept custom regions from the user.

The KILDA toolkit is organized around *kilda.nf*, a Nextflow pipeline. This pipeline can run two subworkflows: (i) *prepare_kmers_DB* to create a list of kmers specific to KIV-2 and the normalization regions and (ii) *kiv2_counts* to count the number of KIV-2 from FASTQ files. The second component uses a Python script, *kilda.py*, to estimate the KIV-2 repeats, which can also be run independently. For ease of use, KILDA is provided with a list of kmers specific to KIV-2 and the *LPA* gene for the normalization region (see the repository link in the “Data availability” section); hence, running the first subworkflow is optional.

The KILDA pipeline is intended to be flexible and the user can decide to run one or both of the subworkflows by setting the corresponding booleans to true or false in the config file.

### Prepare kmers DB

This subworkflow is designed to generate the list of kmers specific to the KIV-2 and normalization regions (Supplementary Fig. S1):

- Kmers from the reference genome are generated with Jellyfish v2.2.10 [13], based on regions given as bed files.
- Kmers are filtered based on their occurrences (one for the normalization kmers and six for the KIV-2 kmers, as there are six KIV-2 repeats in the reference genome).
- The kmers with a count >0 anywhere else are discarded.
- Kmers in common between the KIV-2 and normalization regions are discarded.
- The kmers are written under the FASTA and tsv formats.

This ensures that we have representative kmers that are specific to the KIV-2 and the normalization regions. A template configuration file is provided; users can modify the regions of interest, as well as the kmer size.

### KIV-2 counts

The second subworkflow from KILDA can infer the number of KIV-2 repeats directly from FASTQ files. It relies on kmers specific to the KIV-2 region and to one or more normalization regions to estimate the KIV-2 repeats. These lists of kmers can be produced with the subworkflow described previously.

The kmers from both lists are counted with *jellyfish count* and are written to a tab-delimited counts file with *jellyfish dump*. The counts files are then given as input to *kilda.py* for estimation of the KIV-2 copy numbers (Supplementary Fig. S2).

### kilda.py

The Python script reads the counts files and computes the number of KIV-2 repeats by dividing the mean occurrence of the KIV-2 kmers by the mean occurrence of the normalization kmers. To note, absent kmers are ignored in the computation of the means. A report for each sample, indicating the number of missing kmers and the occurrence means, is printed to the terminal.

Moreover, if the --*rsids* option was set and the user provided a file listing reference and alternative kmers for a list of single nucleotide polymorphisms (SNPs), *kilda.py* will report their occurrences, which can be used to assess their presence or absence in the samples. This can help to give more context around the number of KIV-2 repeats, as some SNPs can affect Lp(a) concentration [4]. If the *--plot* option is activated, KILDA generates a visual representation of the distribution of the occurrences of the KIV-2 and normalization kmers for each sample (Supplementary Fig. S3).

*kilda.py* can be executed independently or from within *kilda.nf*.

### Comparison against DRAGEN-LPA on the 1000 Genomes Project

KILDA was tested against the DRAGEN-LPA caller on the 1000 Genomes Project dataset [14]. The DRAGEN-LPA KIV-2 copy number predictions were obtained from the preprint’s supplementary tables. KILDA’s predictions were computed from the 1000G phase 3 FASTQ files, using a list of 31-mers produced with the *prepare_kmers_DB* subworkflow. Additionally, we compared the output of KILDA directly to the “high-confidence” Bionano KIV-2 alleles available from supplementary table S2 of the DRAGEN-LPA manuscript [12].

In addition to its primary functionality, KILDA can process a predefined list of variants with corresponding kmers, which contain reference and alternative alleles. We thus selected the following variants: *rs10455872* (+5.4 mg/dl), *rs3798220* (+45 mg/dl), and *rs41272114* (−5 mg/dl), with established impact on Lp(a) concentrations [4]. A sample was considered as carrier if at least one alternative kmer was detected in the sample.

### *In silico* predictions

We created *in silico* samples with predetermined KIV-2 copy numbers to test KILDA’s robustness under different scenarios of sequencing coverage and quality.

First, we created a “diploid” reference, by duplicating GRCh38 and applying dbSNP’s SNPs to one of the copies, using *bcftools consensus*. Then, for each condition (coverage between 2× and 30×, and mean read quality between 12 and 28), we created 40 samples by adding a random number of KIV-2 sequences (between 6 and 30) to each of the haplotypes, resulting in samples with KIV-2 copy numbers between 12 and 60. The KIV-2 sequences were randomly selected from the six KIV-2 sequences from GRCh38.

Finally, we generated whole genome sequencing data as paired-end reads of 150 bp, using *bbmap randomreads.sh* with different options regarding coverage and mean quality. For the coverage (option *--coverage*), a fixed mean quality of 28 was set (the default value of *bbmap randomreads.sh*), and we simulated reads with estimated coverages of 2×, 5×, 10×, 20×, and 30×. For the mean quality (option *--midq)*, a fixed coverage of 10× was set, and we generated libraries of reads with a mean quality of 12, 14, 16, 18, 20, and 28.

The *kiv2_counts* subworkflow was run on each set of 40 samples, using a list of 31-mers produced with KILDA. The predicted KIV-2 copy numbers were compared to the known number of KIV-2 induced in the samples.

## Results and discussion

### Comparison against DRAGEN-LPA on the 1000 Genomes Project

**Figure 1.**
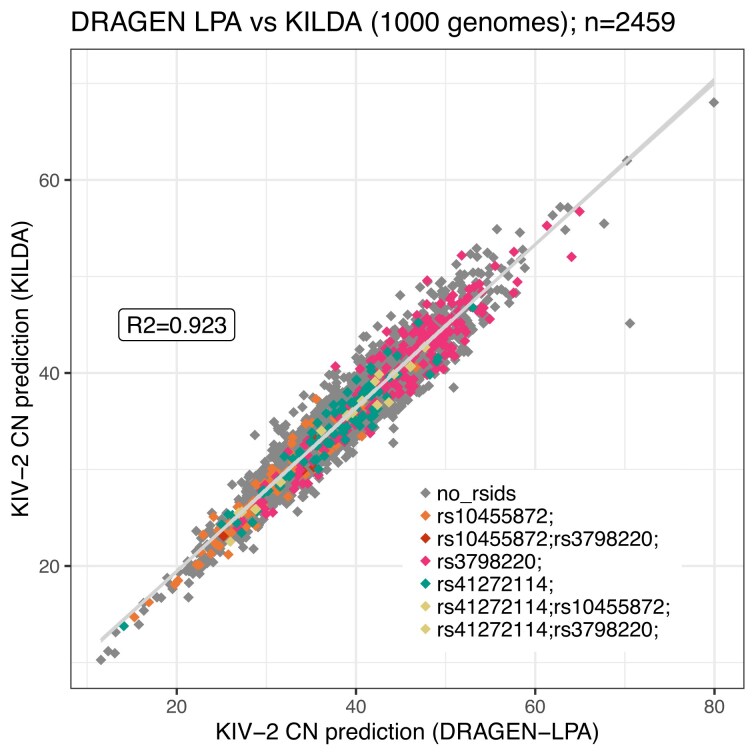
KILDA predictions against DRAGEN-LPA predictions for the number of KIV-2 copies on samples from the 1000 Genomes Project. For KILDA, 31-mers were used to make the predictions. The samples are colored by the presence of variants in samples as detected by KILDA.

The overlap between the 1000 Genomes Project samples processed by both KILDA and DRAGEN-LPA resulted in 2459 samples. Some samples were removed from the 1000 Genomes archive (mostly due to bad QC or contamination). KILDA’s predictions for the 1000 Genomes Project samples were highly correlated to the DRAGEN-LPA predictions with an *R*^2^ = 0.923 (*y* = 1.09*x* − 0.2) (see Fig. 1). However, there was a factor of 1.09 between the predictions made with KILDA, using 31-mers, and DRAGEN-LPA. Testing with different values of *k* showed that increasing the kmer size resulted in a reduction of this factor (Supplementary Fig. S4), at the cost of more computational resources. If reads are of high quality and computational resources are available, we recommend using higher values of *k* when using KILDA. To ensure reliable comparisons between tools, percentiles can be computed instead of raw KIV-2 counts [15]. KILDA’s predictions were also correlated to the “high-confidence” Bionano KIV-2 set from supplementary table S1 of the DRAGEN-LPA manuscript (*R*^2^ = 0.962, *y* = 1.10*x* − 0.36). The comparison of KILDA’s prediction against the optically mapped samples is shown in Supplementary Fig. S5.

Three variants were given as input to KILDA to give more context when interpreting the number of KIV-2 in relation to Lp(a): *rs10455872*,*rs3798220*, and *rs41272114*, which were found in 86, 206, and 99 samples, respectively. These samples overlapped well with the carriers of these SNPs in the 1000 Genomes Project VCF file; detailed counts are available in Supplementary Table S1.

The mean number of KIV-2 repeats was different between the carriers and non-carriers for *rs10455872* (*t*-test: 27.80 versus 35.70, *P*-value <2.2e−16) and *rs3798220* (*t*-test: 39.82 versus 35.03, *P*-value <2.2e−16). Concerning *rs41272114*, the mean number of repeats was similar between carriers and non-carriers (*t*-test: 34.10 versus 35.49, *P*-value = 0.02). Thus, as previously described [4], *rs10455872* is partially tagging smaller isoforms. However, *rs3798220*, an Lp(a)-increasing variant, previously thought to be associated with lower isoforms [4], is present in samples with a higher number of genetic KIV-2, which could be wrongly interpreted as having lower Lp(a) and lower risk of CVD, demonstrating the importance of including the presence of Lp(a)-affecting variants when interpreting the number of KIV-2 copies. The discordance between our results and the literature can be explained when stratifying the results by ancestry. *rs3798220* is indeed present in smaller isoforms in Europeans (*t*-test: 28.94 versus 34.09, *P*-value = 0.02) but associated with longer isoforms in Americans (*t*-test: 39.92 versus 34.41, *P*-value = 2.95e−14), which are the main carriers of this SNP (*n* = 109). In addition, this SNP is rare in Africans (*n* = 5) who have smaller isoforms (Supplementary Fig. S6), highlighting the importance of directly estimating KIV-2 repeats instead of uniquely relying on rsids when studying diverse populations.

The *kiv2_counts* pipeline was run on a Debian 11 server, with five CPUs allocated to each sample. Each sample was processed in 18 min, with a peak memory usage of 830 MB, the counting of the kmers with *jellyfish count* being the most time-consuming (Supplementary Table S2).

### *In silico* predictions

The *R*^2^ between the predicted KIV-2 copy numbers and the copy numbers included in the simulated samples remained higher than 0.99 for coverages between 5× and 30× (Supplementary Fig. S7). For 2×, the lowest tested coverage, the *R*^2^ value was 0.97, demonstrating KILDA’s accuracy even at very low coverages.

Regarding the reads’ mean quality, the *R*^2^ between the predicted and simulated KIV-2 copy numbers was >0.99 for mean sequencing qualities between 18 and 28 (Supplementary Fig. S8). KILDA’s accuracy remained high for libraries with mean qualities of 16 and 14 (*R*^2^ = 0.98 and 0.95, respectively), showing KILDA’s robustness to sequencing libraries of lower quality. KILDA’s predictions started to falter when simulating a library with a mean quality of 12, but realistically, modern sequencing libraries should reach higher mean qualities.

Interestingly, the difference factor of 1.10× observed with DRAGEN-LPA was not present here (*y* = 1*x* − 1).

## Conclusion

Here, we demonstrated the performance of KILDA on a real whole genome sequencing dataset: the 1000 Genomes Project, and against simulated datasets with a known number of KIV-2 repeats. Future work should focus on reducing the factor of prediction observed against DRAGEN-LPA, and on including haplotyping of the KIV-2 copy numbers, as it can influence Lp(a). To further validate KILDA’s utility and applicability in clinical settings, it is imperative to evaluate its performance on whole exome sequencing and targeted panel datasets. This additional testing would help determine whether KILDA can be effectively integrated into routine diagnostic practices. It would therefore provide a free tool complementary to that of variant calling within unmappable regions [16] using short-read sequencing, instead of long-read sequencing [17], which is currently not commonly used in clinical practice. Moreover, KILDA’s predictions based on long-read datasets (PacBio and Nanopore) remain unreliable, due to the current low-accuracy nature of the sequences. We hope that, with the continuous improvement of long-read sequencing, KILDA will soon be able to predict KIV-2 repeats from long-read datasets, which could also include haplotyping.

KILDA is an open-source Nextflow pipeline, which can estimate the number of KIV-2 repeats along with the presence of Lp(a)-affecting variants. One of the main advantages of KILDA is its capacity to estimate KIV-2 numbers based on the *LPA* sequence alone and so it does not need normalization regions across the genome, which are often not captured in clinical practice. KILDA is provided with an Apptainer image, predetermined lists of KIV-2 and normalization kmers, and a file listing kmers corresponding to three important Lp(a)-related SNPs, for ease of use.

KILDA was only tested on KIV-2 repeats; however, its method should theoretically work on any other variable number tandem repeat.

## Supplementary Material

lqaf070_Supplemental_File

## Data Availability

KILDA is publicly available at https://doi.org/10.5281/zenodo.15111514 and https://github.com/HCL-HUBL/KILDA along with a recipe to build an Apptainer image containing all the required dependencies.

## References

[1] Tsao CW, Aday AW, Almarzooq ZI et al. Heart disease and stroke statistics—2023 update: a report from the American Heart Association. Circulation. 2023; 147:e93–621. 10.1161/CIR.0000000000001123. 36695182 PMC12135016

[2] Patel AP, Wang M, Pirruccello JP et al. Lp(a) (lipoprotein[a]) concentrations and incident atherosclerotic cardiovascular disease. Arterioscler Thromb Vasc Biol. 2021; 41:465–74. 10.1161/ATVBAHA.120.315291. 33115266 PMC7769893

[3] Kronenberg F, Mora S, Stroes ESG et al. Lipoprotein(a) in atherosclerotic cardiovascular disease and aortic stenosis: a European Atherosclerosis Society consensus statement. Eur Heart J. 2022; 43:3925–46. 10.1093/eurheartj/ehac361. 36036785 PMC9639807

[4] Coassin S, Kronenberg F Lipoprotein(a) beyond the kringle IV repeat polymorphism: the complexity of genetic variation in the *LPA* gene. Atherosclerosis. 2022; 349:17–35. 10.1016/j.atherosclerosis.2022.04.003. 35606073 PMC7613587

[5] Schmidt K, Noureen A, Kronenberg F et al. Structure, function, and genetics of lipoprotein(a). J Lipid Res. 2016; 57:1339–59. 10.1194/jlr.R067314. 27074913 PMC4959873

[6] Durlach V, Bonnefont-Rousselot D, Boccara F et al. Lipoprotein(a): pathophysiology, measurement, indication and treatment in cardiovascular disease. A consensus statement from the Nouvelle Société Francophone d’Athérosclérose (NSFA). Arch Cardiovasc Dis. 2021; 114:828–47. 10.1016/j.acvd.2021.10.009. 34840125

[7] Friedewald WT, Levy RI, Fredrickson DS Estimation of the concentration of low-density lipoprotein cholesterol in plasma, without use of the preparative ultracentrifuge. Clin Chem. 1972; 18:499–502. 10.1093/clinchem/18.6.499. 4337382

[8] Yeang C, Witztum JL, Tsimikas S Novel method for quantification of lipoprotein(a)-cholesterol: implications for improving accuracy of LDL-C measurements. J Lipid Res. 2021; 62:100053 10.1016/j.jlr.2021.100053. 33636163 PMC8042377

[9] Tromp TR, Ibrahim S, Nurmohamed NS et al. Use of lipoprotein(a) to improve diagnosis and management in clinical familial hypercholesterolemia. Atherosclerosis. 2023; 365:27–33. 10.1016/j.atherosclerosis.2022.11.020. 36473758

[10] Nordestgaard BG, Langsted A Lipoprotein(a) and cardiovascular disease. Lancet. 2024; 404:1255–64. 10.1016/S0140-6736(24)01308-4. 39278229

[11] Dron JS, Wang M, Patel AP et al. Genetic predictor to identify individuals with high lipoprotein(a) concentrations. Circ Genom Precis Med. 2021; 14:1255–64. 10.1161/CIRCGEN.120.003182. PMC7887018 33522245

[12] Behera S, Belyeu JR, Chen X et al. Identification of allele-specific KIV-2 repeats and impact on Lp(a) measurements for cardiovascular disease risk. BMC Med Genomics. 2024; 17:255 10.1186/s12920-024-02024-0. 39449055 PMC11515395

[13] Marçais G, Kingsford C A fast, lock-free approach for efficient parallel counting of occurrences of *k*-mers. Bioinformatics. 2011; 27:764–70. 10.1093/bioinformatics/btr011. 21217122 PMC3051319

[14] Auton A, Abecasis GR, Altshuler DM et al. A global reference for human genetic variation. Nature. 2015; 526:68–74. 26432245 10.1038/nature15393 PMC4750478

[15] Szarek M, Reijnders E, Jukema JW et al. Relating lipoprotein(a) concentrations to cardiovascular event risk after acute coronary syndrome: a comparison of 3 tests. Circulation. 2024; 149:192–203. 10.1161/CIRCULATIONAHA.123.066398. 37632469 PMC10782942

[16] Di Maio S, Zöscher P, Weissensteiner H et al. Resolving intra-repeat variation in medically relevant VNTRs from short-read sequencing data using the cardiovascular risk gene LPA as a model. Genome Biol. 2024; 25:167 10.1186/s13059-024-03316-5. 38926899 PMC11201333

[17] Amstler S, Streiter G, Pfurtscheller C et al. Nanopore sequencing with unique molecular identifiers enables accurate mutation analysis and haplotyping in the complex lipoprotein(a) KIV-2 VNTR. Genome Med. 2024; 16:117 10.1186/s13073-024-01391-8. 39380090 PMC11462820

